# Role of microRNAs in regulation of doxorubicin and paclitaxel responses in lung tumor cells

**DOI:** 10.1186/s13008-023-00093-8

**Published:** 2023-07-21

**Authors:** Amirhosein Maharati, Meysam Moghbeli

**Affiliations:** 1 Student Research Committee, Faculty of Medicine, Mashhad University of Medical Sciences, Mashhad, Iran; 2 Department of Medical Genetics and Molecular Medicine, School of Medicine, Mashhad University of Medical Sciences, Mashhad, Iran

**Keywords:** Lung cancer, Paclitaxel, Doxorubicin, Chemo resistance, microRNA

## Abstract

Lung cancer as the leading cause of cancer related mortality is always one of the main global health challenges. Despite the recent progresses in therapeutic methods, the mortality rate is still significantly high among lung cancer patients. A wide range of therapeutic methods including chemotherapy, radiotherapy, and surgery are used to treat lung cancer. Doxorubicin (DOX) and Paclitaxel (TXL) are widely used as the first-line chemotherapeutic drugs in lung cancer. However, there is a significant high percentage of DOX/TXL resistance in lung cancer patients, which leads to tumor recurrence and metastasis. Considering, the side effects of these drugs in normal tissues, it is required to clarify the molecular mechanisms of DOX/TXL resistance to introduce the efficient prognostic and therapeutic markers in lung cancer. MicroRNAs (miRNAs) have key roles in regulation of different pathophysiological processes including cell division, apoptosis, migration, and drug resistance. MiRNA deregulations are widely associated with chemo resistance in various cancers. Therefore, considering the importance of miRNAs in chemotherapy response, in the present review, we discussed the role of miRNAs in regulation of DOX/TXL response in lung cancer patients. It has been reported that miRNAs mainly induced DOX/TXL sensitivity in lung tumor cells by the regulation of signaling pathways, autophagy, transcription factors, and apoptosis. This review can be an effective step in introducing miRNAs as the non-invasive prognostic markers to predict DOX/TXL response in lung cancer patients.

## Background

Lung cancer is the most frequent cancer and is responsible for the highest number of cancer-related deaths in the world [1]. Despite the recent progresses in molecular targeted therapies and surgical techniques, the overall 5-years survival rate is still around 15% for lung cancer patients [2]. Non-small cell lung cancer (NSCLC) is the most frequent lung tumor type accounting for 85% of all cases [1, 3]. Targeted drugs have efficient therapeutic benefits for NSCLC patients; however, drug resistance is a frequent challenge that is finally observed among a large proportion of NSCLC patients [4, 5]. Cisplatin, carboplatin, paclitaxel, docetaxel, gemcitabine, and pemetrexed are the frequently used chemotherapeutic options for NSCLC patients [6–8]. DNA-damaging factors are the most commonly used types of chemotherapeutic drugs [9, 10]. They prevent cell proliferation while induce cell death by the suppression of the double-strand breaks rejoining [11]. Microtubule targeting agents (MTAs) are also conventional chemotherapeutic drugs for NSCLC patients. They bind to microtubules at various sites to disrupt their dynamics and structure, resulting in cell cycle arrest and subsequent cell death [12, 13]. Doxorubicin is considered as an inhibitor of DNA synthesis and transcription by targeting topoisomerase II that results in cell cycle arrest and apoptosis [14, 15]. The combination of doxorubicin with other chemotherapeutic drugs is a standard therapeutic regimen for lung carcinoma [16]. Nevertheless, the emergence of drug resistance has impaired its efficacy as a therapeutic agent [17]. Doxorubicin as an anthracycline is frequently used to treat SCLC patients. Despite more than half of the patient’s response to drug, the median survival of SCLC patients is approximately 10–12 months in primary tumor stage [18–20]. Paclitaxel (PTX) is a critical therapeutic option for the advanced stage NSCLC [21]. It functions by binding to the β subunit of tubulin to inhibit the establishment of microtubules that results in cell cycle disruption and apoptosis [22]. However, the development of resistance to paclitaxel leads to treatment failure and reduced survival rates for patients. As the poor prognosis is associated with advanced stages and drug resistance in lung cancer, it is required to introduce the novel diagnostic and prognostic biomarkers to improve the therapeutic strategies in these patients. MiRNAs are small non-coding RNAs that are found in all eukaryotic cells and play a vital role in post-transcriptional inhibition of target mRNAs [23, 24]. MiRNAs have pivotal roles in lung tumor progression by regulation of various cellular processes such as cell proliferation, angiogenesis, and epithelial-mesenchymal transition (EMT) [25, 26]. They are implicated in drug resistance by affecting various cellular processes such as cell survival, apoptosis, angiogenesis, and migration [27]. MiRNAs deregulations are associated with chemo resistance in various cancers [28, 29]. Therefore, in the present review we discussed the role of miRNAs in DOX/TXL responses in lung tumor cells to introduce them as the probable non-invasive prognostic markers in lung cancer patients (Table 1). Web of Science, Embase, PubMed, Cochrane Library, and Google scholar were searched and assessed until the May 2023 without language limitations. The reference lists were also manually searched for the relevant publications including the review articles and original researches. The search strategy was based on “microRNA”, “Doxorubicin”, “Paclitaxel”, “Drug resistance”, and “Lung cancer” keywords.

**Table 1:** Role of miRNAs in regulation of DOX and TXL responses in lung tumor cells

miRNA	Target	Samples	Results	Clinical application	Study	Year
Signaling pathways						
miR-7	EGFR	20 T 20N* A549, H1395, 95C and 95D cell lines	Increased Paclitaxel sensitivity	Diagnosis	Liu [39]	2014
miR-4262	PTEN	20 T 20N A549, H1299, A549/PTX and H1299/PTX cell lines Xenograft model	Increased Paclitaxel resistance	Diagnosis	Sun [42]	2019
miR-181a	PTEN	A549, A549/PTX, and A549/DDP cell lines	Increased Paclitaxel resistance	Diagnosis	Li [43]	2015
miR-4715-5p	RAC1	25 T 25N A549, Calu1, H1299, and HOP62 cell lines Xenograft model	Increased Paclitaxel sensitivity	Diagnosis	Yang [52]	2019
miR-9600	STAT3	144 T 20N A549, SPC-A-1, H1299, SK-MES-1, NCI-H520, 95D and 16HBE cell lines Xenograft model	Increased Paclitaxel sensitivity	Diagnosis and prognosis	Sun [57]	2016
miR-1247-3p	STAT5A	162 T 162N NCI-H1299, NCI-H1395, A549, NCL-H460, PG49, NCI-H1993 cell lines Xenograft model	Increased Doxorubicin sensitivity	Diagnosis and prognosis	Lin [59]	2022
miR-337-3p	RAP1A	H1155, H1299, H1819, H1993, HCC2935, and HCC515 cell lines	Increased Paclitaxel sensitivity	Diagnosis	Du [67]	2012
miR-34c	NOTCH1	30 T 30N A549, H1299, and 293 T cell lines Xenograft model	Increased Paclitaxel sensitivity	Diagnosis	Yang [69]	2020
Transcription factors and DNA binding proteins						
miR-138	ZEB2	A549, NCI-H23, A549/ADM and NCI-H23/ADM cell lines	Increased Doxorubicin sensitivity	Diagnosis	Jin [73]	2016
miR-194-5p	HIF-1	H460 and A549 cell lines	Increased Doxorubicin sensitivity	Diagnosis	Xia [75]	2021
mR-608	TFAP4	37 T 37N 96 T serum 136N serum A549 and HCC4006 cell lines	Increased Doxorubicin sensitivity	Diagnosis	Wang [81]	2019
miR-935	SOX7	30 T 30N A549 cell line	Increased Paclitaxel resistance	Diagnosis	Peng [88]	2018
miR-30c	MTA1	A549 and H460 cell lines	Increased Paclitaxel sensitivity	Diagnosis	Lu [97]	2017
miR- 137	NUCKS1	50 T 50N A549, A549/PTX and A549/CDDP cell lines Xenograft model	Increased Paclitaxel sensitivity	Diagnosis and prognosis	Shen [104]	2016
Structural factors						
miR-200c	CTSL	A549 and A549/TAX cell lines	Increased Paclitaxel sensitivity	Diagnosis	Zhao [114]	2018
miR-421	KEAP1	129 T 129N 10 T serum 10N serum A549, H358, H1650, H460, and H1975 cell lines Xenograft model	Increased Paclitaxel resistance	Diagnosis and prognosis	Duan [119]	2019
miR-223	FBW7	A549, NCI-H358, NCI-H1299 and HCC827 cell lines	Increased Doxorubicin resistance	Diagnosis	Li [126]	2016
miR-490-5p	UBE2T	50 T (20R 30S) 50N H1299 and A549 cell lines Xenograft model	Increased Paclitaxel sensitivity	Diagnosis	Wang [129]	2023
miR-558	MMP1/MMP17	46 T 46N A549, H1299, H358, and PC9 cell lines	Increased Paclitaxel resistance	Diagnosis	Li [131]	2021
miR-197-3p	p120-ctn	326 T 326N A549, H1299, H460 and SPC-A-1 cell lines Xenograft model	Increased Paclitaxel and Doxorubicin sensitivity	Diagnosis and prognosis	Yang [137]	2019
miR-708-5p	COX-2/mPGES-1	A549, A549-ER, and A549-PR cell lines	Increased Paclitaxel sensitivity	Diagnosis	Monteleone [143]	2020
miR-486-3p	CRABP2	65 T (30R 35S) 65N A549 and H1299 cell lines Xenograft model	Increased Paclitaxel sensitivity	Diagnosis	Wu [144]	2022
miR-526b-5p	GRK5	65 T 65N A549, H3122, H1975, and H2342 cell lines Xenograft model	Increased Paclitaxel sensitivity	Diagnosis	Liu [146]	2021
mR-299-3p	ABCE1	20 T 20N NCI-H69 cell line	Increased Doxorubicin sensitivity	Diagnosis	Zheng [149]	2015
Apoptosis and DNA repair						
miR-1273f	MDM2	20 T 20N A549 and A549/Taxol cell lines Xenograft model	Increased Paclitaxel sensitivity	Diagnosis and prognosis	Xu [153]	2021
miR-107	Bcl-w	A549 and HEK 293 T cell lines Xenograft model	Increased Paclitaxel sensitivity	Diagnosis	Lu [161]	2017
miR-30a-5p	BCL-2	94 T 94N A549, H460, A549/PR, and H460/PR cell lines Xenograft model	Increased Paclitaxel sensitivity	Diagnosis and prognosis	Xu [162]	2017
miR-7-5p	PARP1	H69, H69AR, and H446AR	Increased Doxorubicin sensitivity	Diagnosis	Lai [167]	2019
miR-195	CHEK1	57 T 57N H1155, H1993 and H358 cell lines Xenograft model	Increased Paclitaxel sensitivity	Diagnosis and prognosis	Yu [169]	2018
miR-433-3p	CHEK1	41 T 41N A549, H1299, A549/PTX and H1299/PTX cell lines	Increased Paclitaxel sensitivity	Diagnosis	Jin [170]	2022
Autophagy and drug efflux						
miR-17-5p	Beclin1	A549, H596, A549-T24, and H596-TxR cell lines	Increased Paclitaxel sensitivity	Diagnosis	Chatterjee [180]	2014
miR-199a-5p	ATG5	A549, H1299, H661, H522, H1944, and A549/T cell lines	Increased Paclitaxel resistance	Diagnosis	Zeng [183]	2021
miR-155	AKT/ERK	A549 and A549/dox cell lines	Increased Doxorubicin resistance	Diagnosis	Lv [189]	2016

*Tumor (T) and normal (N) tissues

### Signaling pathways

**Fig. 1:**
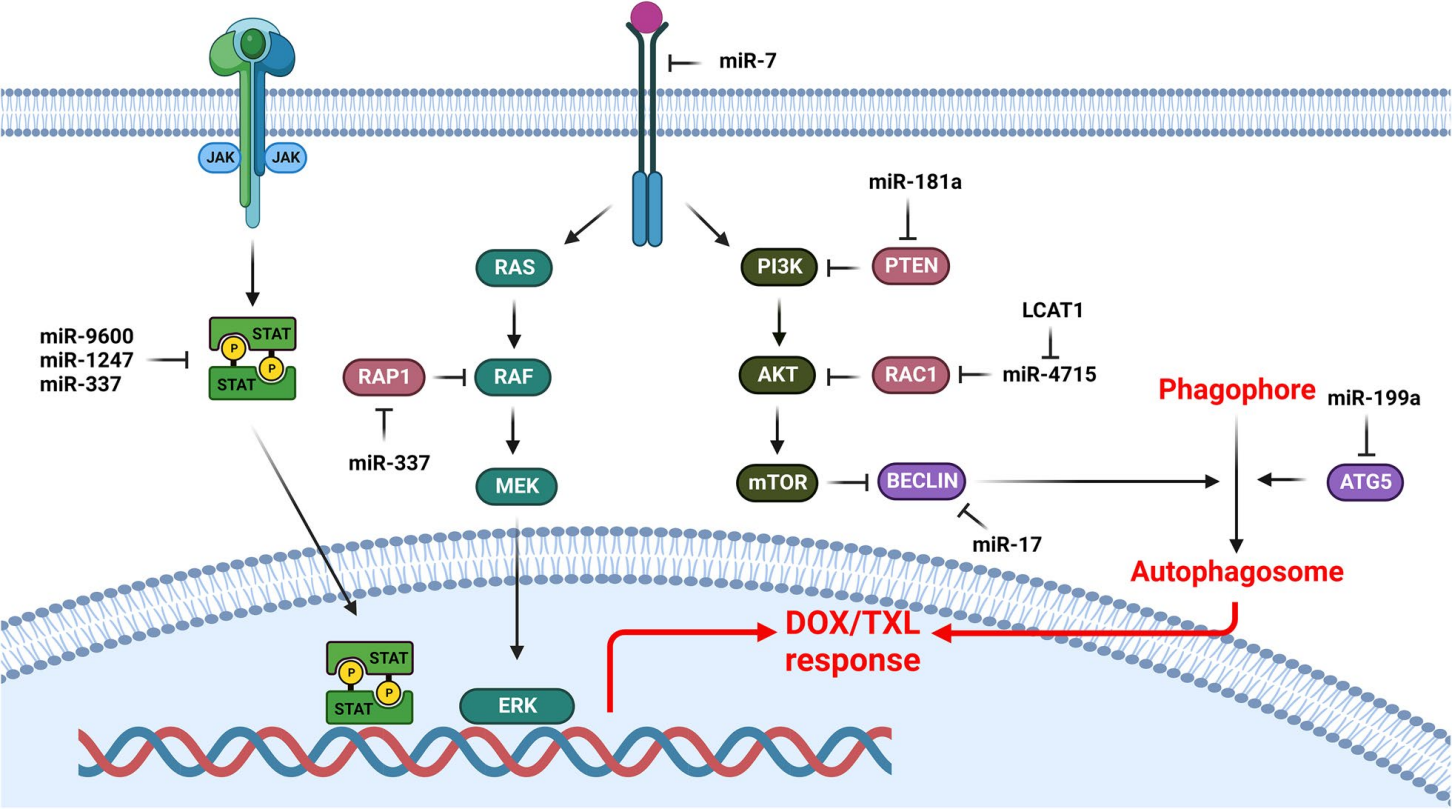
Role of miRNAs in DOX/TXL responses via regulation of signaling pathways and autophagy in lung tumor cells. (Created with BioRender.com)

MiRNAs are involved in DOX/TXL response of lung tumor cells via the regulation of signaling pathways (Fig. 1). PI3K/AKT is one of the main oncogenic signaling pathways that is directly associated with the extracellular growth factors. It is mainly triggered by the activation of receptor tyrosine kinases (RTKs) that subsequently activates PI3K/AKT/mTOR axis [30]. EGFR belongs to the RTK protein family that has a key role in cell proliferation by activation of PI3K/AKT and MAPK signaling pathways. MiR-7 attenuated NSCLC progression via targeting several oncogenes, such as PAK1, EGFR, RAF1, IRS1, and IRS2 that resulted in inhibition of the EGFR/AKT axis [31–34]. Activation of EGFR downstream pathways, including STAT, PI3K/AKT, and MAKP intensifies the chemo resistance of tumor cells [32, 34–38]. It has been reported that miR-7 increased the PTX sensitivity via EGFR targeting in NSCLC cells [39]. PTEN is a negative regulator of the PI3K/Akt axis and is frequently down regulated or mutated in lung cancer [40, 41]. MiR-4262 promoted PTX resistance through PTEN targeting and subsequent PI3K/AKT activation in NSCLC cells [42]. MiRNA-181a has been identified as a contributor to the acquisition of EMT, as well as increased invasion and migration in lung adenocarcinoma cells through PTEN targeting. MiR-181a also increased the sensitivity of cancer cells to paclitaxel treatment [43]. Reactive oxygen species (ROS) is involved in VEGF induced activation of the PI3K/AKT axis [44, 45]. Rac1 belongs to the Rho protein family that regulates growth factors and cytokines [46]. P21-activated kinase (PAK1) is a ser/thr kinase that interacts with Rac1 and Cdc42 [47]. EGF promotes tumor cell migration by Rac1 mediated activation of PI3K/Akt and PAK1 [48]. Long noncoding RNAs (lncRNAs) are a class of non-coding RNAs that have pivotal roles in regulation of cell growth, angiogenesis, survival, and motility [49–51]. The significant up regulation of LCAT1 has been reported in lung cancer tissues that were associated with unfavorable prognosis. LCAT1 enhanced the lung tumor growth through the miR-4715-5p/RAC1 axis. The reduction of RAC1 activity hindered the cell proliferation and mobility and its function was regulated by PAK1. Both RAC1 and PAK1 were found to be reduced in cells with elevated levels of miR-4715-5p and in cells where LCAT1 was silenced. EHop-016 as a Rac GTPase inhibitor reduced the viability of lung tumor cells. The efficacy of EHop-016 and paclitaxel in treating lung cancer cells was improved when they were used in combination. EHop-016 as an adjuvant therapy enhanced the paclitaxel efficacy in lung cancer patients who had LCAT1 up regulation [52].

JAK/STAT pathway has a critical role in cell proliferation, inflammation, and apoptosis. IL-6 activates the JAK2 that promotes the STAT3 dimerization and nuclear transportation to regulate the JAK/STAT target genes [53]. STAT3 is a key regulator of cancer-related inflammation and tumor progression [54]. It promotes tumor cell growth, invasion, immunosuppression, angiogenesis, and drug resistance [55]. STAT3 also promotes tumorigenesis by inhibiting cell death via Bcl-xL and Bcl-2 up regulations [56]. MiR-9600 enhanced paclitaxel sensitivity of NSCLC through targeting STAT3 that resulted in CDK2, CCND1, cyclin E, and p-RB down regulations [57]. STAT5A is a transcription factor that participates in cell proliferation, migration, and aggressiveness [58]. MiR-1247-3p has been reported to be down regulated in lung adenocarcinoma tissues that were associated with advanced stages and metastatic tumors. It suppressed Doxorubicin resistance in lung tumor cells via STAT5A targeting [59]. RAP1A as one of the RAP1 isoforms is involved in regulation of microtubule dynamics. RAP1 triggers the MAPK/ERK axis and phosphorylates microtubule-associated proteins such as MAP2 and MAP4 [60–64]. It can also regulate the paclitaxel sensitivity of tumor cells via extracellular matrix and cell interactions [65, 66]. MiR-337-3p increased the paclitaxel sensitivity of lung tumor cells via STAT3 and RAP1A targeting. STAT3 antagonized microtubule depolymerization by binding to stathmin, while RAP1A suppressed microtubule polymerization via triggering ERK/MAPK and MAP2 and MAP4 phosphorylations. Depletion of RAP1A or STAT3 disrupted normal microtubule dynamics that sensitized tumor cells toward the microtubule-targeting agents. Therefore, paclitaxel treatment and RAP1A/STAT3 down regulation synergistically disrupted microtubule function, resulting in G2/M arrest and cell death [67].

NOTCH is a developmental signaling pathway that has critical roles in embryogenesis and tumor progression. It can be triggered by activation of NOTCH receptors that releases the NICD into the cytoplasm. Subsequently, NICD enters into the nucleus and regulate the NOTCH target genes by MAML/CSL transcriptional machinery [68]. There was significant miR-34c down regulation in NSCLC tissues. MiR-34c sensitized the NSCLC cells to paclitaxel and cisplatin through the NOTCH1 targeting [69].

### Transcription factors and DNA binding proteins

**Fig. 2:**
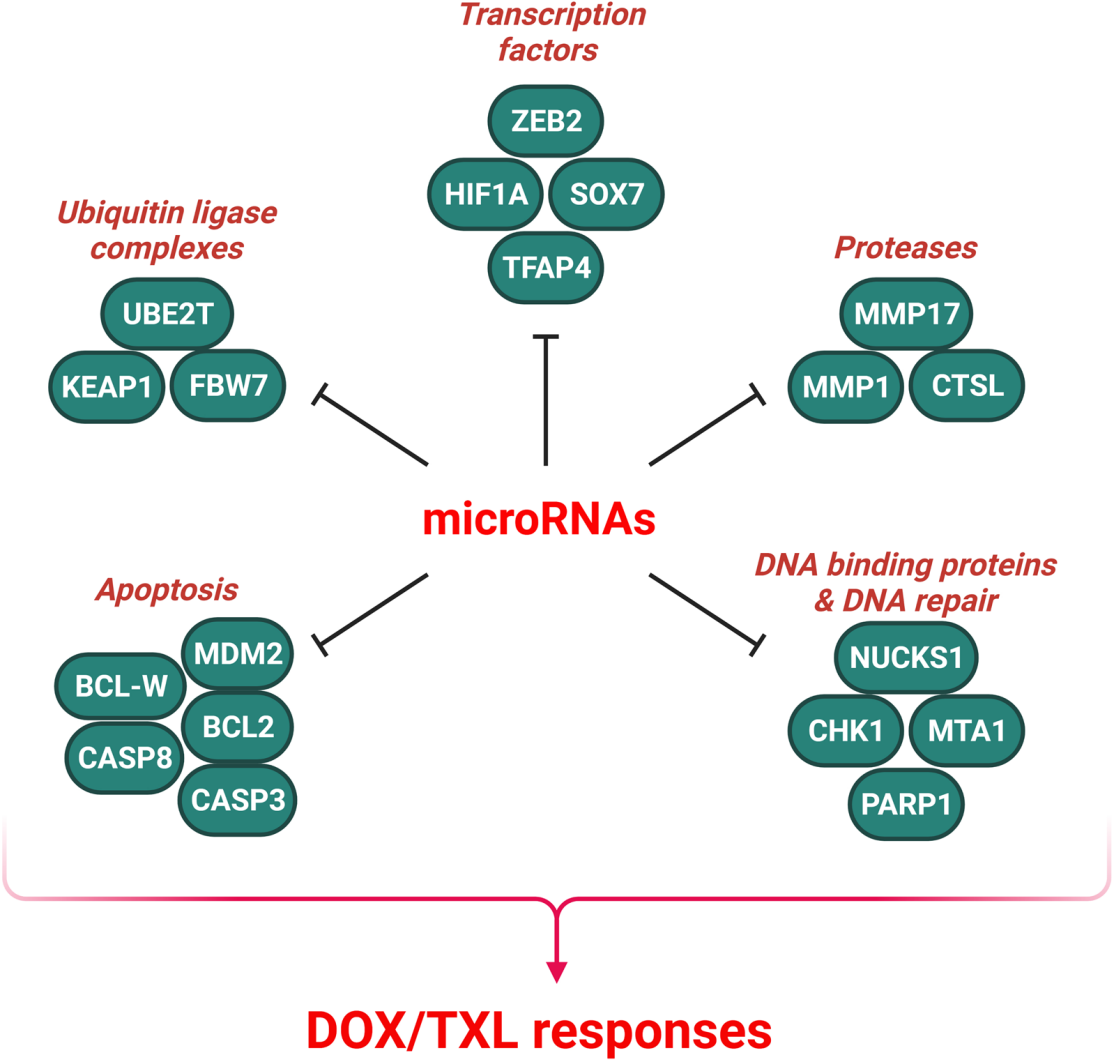
Role of miRNAs in DOX/TXL responses via regulation of transcription factors, ubiquitination, proteases, and DNA repair in lung tumor cells. (Created with BioRender.com)

MiRNAs are involved in DOX/TXL response of lung tumor cells via the regulation of transcription factors and DNA binding proteins (Fig. 2). EMT has a key role in NSCLC progression and chemotherapy response, by which tumor cells lose their epithelial features and acquire a mesenchymal and aggressive phenotype [70]. ZEB2 belongs to the zinc finger homeobox protein family that regulates the tumor progression and chemotherapy response [71]. ZEB2 suppresses the CDH1 to promote tumor cell invasion and chemo resistance. However, the inhibition of ZEB2 by several miRNAs can effectively reverse this effect and lead to the suppression chemo resistance [72]. There was miR-138 down regulation in chemo resistant NSCLC cells. MiR-138 up regulated the E-cadherin while down regulated the Vimentin to sensitize NSCLC cells to DOX via ZEB2 targeting [73]. HIF1A as a basic helix-loop-helix protein is the master regulator of hypoxia response that mediates drug resistance via up regulation of P-glycoprotein (P-gp) [74]. There was significant down regulation of miR-194-5p in yypoxia-induced DOX-resistant NSCLC cells. MiR-194-5p directly targeted HIF-1, which subsequently impaired the expression of downstream proteins, such as P-gp, to enhance the sensitivity of NSCLC cells to DOX. In addition, miR-194-5p regulated the expression of several apoptotic proteins such as PARP and BAX that increased DOX-mediated apoptosis of NSCLC cells [75]. TFAP4 is a transcription factor that is involved in progression of various human cancers [76–79]. It promotes tumor cell proliferation and metastasis, while represses the cell death [79, 80]. It has been shown to activate the Wnt/β-catenin pathway to enhance hepatocellular carcinoma progression [80]. There was miR-608 down regulation in NSCLC samples. MiR-608 facilitated doxorubicin mediated apoptosis in NSCLC cells by targeting TFAP4 [81]. SOX7 is a transcription factor that regulates the cell differentiation, proliferation, migration, and apoptosis and acts as a tumor suppressor in different cancers [82, 83]. In lung cancer, reduced expression of SOX7 is associated with an unfavorable prognosis [84]. Additionally, SOX7 physically interacts with β-catenin and transcription factor 4 to inhibit the Wnt pathway and stemness [85]. Down regulation of SOX7 promotes tumor cell stemness and chemo-resistance [86]. PI3K/Akt axis is a key regulator of cell migration, growth, death, and blood vessel formation [87]. MiR-935 silencing increased paclitaxel mediated apoptosis in NSCLC cells by SOX7 targeting. This intervention down regulated Bcl-2 and p-AKT while up regulated Bax [88]. MTA1 is a member of chromatin remodeling complexes that has key roles in nucleosome remodeling and transcriptional regulation [89]. Curcumin inhibits the tumor cell growth while promotes the programmed cell death [90–92]. It has been shown that Curcumin functions as an anti-tumor drug by modulating signaling pathways, transcription factors, and miRNAs [93–96]. According to a recent investigation, Curcumin enhanced the response of NSCLC cells to Paclitaxel by MTA1 down regulation following the miR-30c-5p up regulation [97]. NUCKS1 is a DNA-binding protein that is a nuclear substrate for DNA-activated Kinase, CDK1, and CK2 [98–101]. It has a key role in regulation of cell cycle progression and transcription during rapid cell growth [102, 103]. MiR-137 promoted PTX sensitivity through NUCKS1 targeting in lung tumor cells [104].

### Structural factors

Cathepsin L (CTSL) belongs to the papain-like cysteine protease family that is associated with the tumor progression [105–107]. It has a crucial role in the various activities of tumor cells, including cell proliferation, migration, viability, invasion, and drug resistance [108–110]. CTSL as an EMT regulator alters the aggressiveness and migration of tumor cells [111]. CTSL also affects drug resistance via EMT-associated transcription factors, such as ZEB1, ZEB2, Slug, and Snail [112]. EMT is regulated by several transcription factors such as Twist, ZEB1, ZEB2, and Snail/Slug [113]. MiRNA-200c suppression reduced paclitaxel sensitivity in lung tumor cells via the up regulation of EMT-related transcription factors. MiRNA-200c inhibited EMT and subsequently improved the response to paclitaxel in lung tumor cells through CTSL targeting [114].

MiRNAs are involved in DOX/TXL response of lung tumor cells via the regulation of protein ubiquitination (Fig. 2). ROS is implicated in both targeted-therapy resistance and chemical resistance that introduce the redox pathway as a reliable tumor therapeutic target [21–25]. KEAP1 acts as an adaptor for substrates by attaching to the CuI3-containing E3 ubiquitin ligase and destructing them through the proteasome pathway [115]. KEAP1 is modified due to ROS-induced oxidative stress, which releases Nrf2 from the KEAP1-Cul3 E3 ligase complex [116]. Subsequently, Nrf2 moves into the nucleus and binds to the antioxidant response element along with a small-Maf binding partner [117, 118]. KEAP1 down regulation was contributed to paclitaxel resistance in NSCLC through the up regulation of miR-421. β-catenin mediated transcription also up regulated the miR-421 [119]. EMT is a multifaceted and reversible process that induces a mesenchymal morphology while reduces the epithelial cell adhesion [120]. FBW7 functions as the substrate recognition component in SCF E3 ligase complex [121]. FBW7 modulates several oncoproteins, including c-Myc, c-Jun, Notch, and CCNE1 [122, 123]. F-box proteins are involved in EMT by modulating inducers and transcription factors [124, 125]. The miR-223/FBW7 axis has been found to enhance doxorubicin sensitivity by regulating EMT in NSCLC cells. Doxorubicin treatment induced EMT in NSCLC cells, but knockdown of Twist hindered this transition through CDH1 up regulation and Vimentin down regulation. Moreover, hypoxia-induced EMT and increased resistance to doxorubicin was accompanied by the reduced levels of FBW7 and E-cadherin while increased Vimentin expression [126]. UBE2T functions as a E2 ubiquitin-conjugating enzyme that catalyzes the ubiquitination of FANCD2 in DNA damage response [127]. Circular RNAs (circRNAs) are a type of non-coding RNA with a stable covalently closed-loop structure [128]. Circ_0092887 inhibiting decreased cell growth and migration, while increased apoptosis in NSCLC cells treated with PTX. Circ_0092887 regulated the PTX resistance via miR-490-5p/UBE2T axis in NSCLC [129]. Matrix metalloproteinases (MMPs) are the key enzymes that break down extracellular matrix (ECM) and collagen to promote tumor angiogenesis and metastasis [130]. Circ_0030998 reduced Taxol resistance by miR-558/MMP1 and MMP17 axes in lung tumor cells [131]. The p120-catenin (p120-ctn) interacts with EMT marker E-cadherin to enhance the lung tumor cell proliferation [132]. It has a pivotal role in modification of the intercellular adhesion and EMT process by interaction with E-cadherin [133–135]. It also bound to cellular structures such as microtubules and cytocentrum to suppress the cell proliferation [136]. There was MALAT1 up regulation in NSCLC tissues that was correlated with poor survival. MALAT1 was associated with resistance to chemotherapeutic drugs such as TXL, gefitinib, DOX, and CDDP. It promoted the cell growth and survival while induced the EMT process in NSCLC cells via miR-197-3p/p120-ctn pathway [137]. ITGB8 is a fibronectin receptor that is involved in cell-cell interactions. There were circDNER up regulations in tumor tissues and plasma exosomes of lung cancer patients. It also promoted the paclitaxel resistance through the miR-139-5p sponging and subsequent ITGB8 up regulation in lung tumor cells [138].

Arachidonic Acid (AA) pathway regulates the cell proliferation, immunity, and homeostasis [139]. COX-1 or COX-2 convert free cytosolic AA to PGH2 [140]. PGE2 has a critical oncogenic role via activation of PI3K/AKT, MAPK, β-catenin, and NF-kB signaling pathways [141, 142]. CHOP is a member of the C/EBP transcription factors involved in adipogenesis and erythropoiesis. Chemotherapy up regulated miR-708-5p while down regulated the AA pathway in lung tumor cells. CHOP and p53 were the transcription factors involved in regulation of chemotherapeutic-mediated miR-708-5p expression. MiR-708-5p also up regulated the p53 and CHOP via a positive feedback loop. There was COX-2 up regulation while miR-708-5p down regulation in paclitaxel resistant lung tumor cells. MiR-708-5p played a tumor suppressive role by COX-2, mPGES-1, and Survivin targeting that resulted in immune evasion [143]. CRABP2 is a retinoic acid binding protein that functions as a cytosol-to-nuclear shuttle to facilitate RA nucleus transfer. Circ_0011298 promoted Taxol resistance via miR-486-3p/CRABP2 axis in NSCLC cells [144].

G protein-coupled receptor kinase 5 (GRK5) belongs to the serine/threonine kinase protein family that is involved in sensing various internal stimuli and regulation of the subsequent signaling pathways [145]. There was circ_0001821 up regulation in NSCLC tissues that was correlated with poor prognosis. Circ_0001821 blocking inhibited the TAX resistance, colony formation, and tumor proliferation via miR-526b-5p/GRK5 axis in NSCLC cells [146]. ABCE1 is a protein that belongs to the ATP-binding cassette (ABC) family and suppresses the RNase L as and interferon-induced nuclease in mammalian cells. ABCE1 is a potential tumor suppressor that is involved in regulation of cell proliferation and apoptosis [147, 148]. It has been indicated that miRNA-299-3p enhanced the doxorubicin-sensitivity in lung cancer via targeting ABCE1. There was miR-299-3p down regulation in doxorubicin-resistant lung tumor tissues compared with the sensitive tissues [149].

### Apoptosis and DNA repair

Tumor cells develop paclitaxel resistance through the various processes such as increased DNA repair, cell cycle regulation, and anti-apoptotic pathways [150–152]. MiRNAs are involved in DOX/TXL response of lung tumor cells via the regulation of apoptosis (Fig. 2). Mouse double minute 2 homolog (MDM2) is an E3 ubiquitin ligase that has a key role in p53 inhibition. There was circ_0002874 up regulation in NSCLC tissues that was correlated with higher stages. Although, there was MDM2 down regulation in NSCLC tissues compared with normal counterparts, increased expression of MDM2 was associated with TNM staging. Circ_0002874 induced paclitaxel resistance by miR-1273f/MDM2 axis in NSCLC cells [153]. Bcl-w belongs to the BCL2 family that blocks apoptosis and promotes cell proliferation [154, 155]. Bcl-w enhances tumor progression by targeting pro-apoptotic factors such as Bax and Bak [156, 157]. Bcl-w deregulation is significantly associated with various types of cancers [158–160]. MiR-107 down regulation was associated with paclitaxel resistance in NSCLC. MiR-107 reduced the levels of p-Akt and p-GSK3β, which were restored by Bcl-w. MiR-107/Bcl-w axis regulated paclitaxel resistance via the PI3K-Akt pathway. MiR-107 increased paclitaxel sensitivity by regulation of Bcl-w expression and PI3K/Akt pathway in NSCLC cells [161]. MiR-30a-5p increased the sensitivity of NSCLC cells to paclitaxel by suppressing BCL-2 and promoting apoptosis. There was a correlation between the miR-30a-5p up regulation and a positive response to paclitaxel treatment in NSCLC patients [162].

MiRNAs are involved in DOX/TXL response of lung tumor cells via the regulation of DNA repair factors (Fig. 2). PARP1 has a key role in DNA repair and gene transcription [163, 164]. DNA damage activates PARP1, which polymerizes ADP-ribose units to recruit the DNA repair proteins in DNA damage location [165]. Homologous recombination (HR) is essential to preserve the genomic stability and chemotherapy response that can be regulated by PARP1 [166]. It has been shown that SCLC cells utilized the miR-7-5p-mediated HR repair by PARP1 targeting to increase the doxorubicin resistance. MiR-7-5p down regulated the BRCA1 and Rad51 in DOX-resistant SCLC cells via PARP1 targeting [167]. Checkpoint kinase 1 (CHK1) is a ser/thr kinase that is involved in regulation of DNA damage and cell cycle response [168]. It promotes cell cycle arrest, DNA repair, and apoptosis. MiR-195 sensitized NSCLC cells to paclitaxel and targeted CHEK1 to modulate the effectiveness of MTAs [169]. Circ_0011292 was up regulated in PTX-resistant NSCLC cells. Depletion of circ_0011292 increased the sensitivity to PTX, suppressed cell growth, aggressiveness, and migration, while induced apoptosis in PTX-resistant NSCLC cells. Circ_0011292 was also contributed to PTX resistance via targeting the miR-433-3p/CHEK1 axis [170].

### Autophagy and drug efflux

Multidrug resistance (MDR) is the ability of tumor cell to resist against the chemotherapy drugs [171]. MDR is acquired through several mechanisms, such as up regulation of ABC transports, inhibition of apoptosis, hypoxia, autophagy, DNA repair, miRNA regulation, and epigenetic changes [172]. Autophagy is a defensive mechanism in tumor cells toward the chemotherapeutic treatment. Chemotherapy mediated autophagy supports the tumor cell metabolism through the recycling of damaged organelles and proteins to prevent DNA damage [173, 174]. Autophagy breaks down damaged cellular components using a lysosomal degradation pathway [175, 176]. This process improves the tumor cell resistance toward apoptosis, hypoxia, and other stress responses, which is essential for MDR [177, 178]. MiRNAs are involved in DOX/TXL response of lung tumor cells via the regulation of autophagy (Fig. 1). Beclin1 is one of the components of autophagy process that facilitates the autophagosomal membrane formation [179]. MiR-17-5p was down regulated in paclitaxel-resistant lung cancer cells, and its up regulation enhanced the paclitaxel response. Inhibition of miR-17-5p ameliorated Beclin1 levels and autophagy, which protected cells against paclitaxel-induced apoptosis. MiR-17-5p-mediated autophagy and paclitaxel treatment also triggered ROS and induced apoptosis in A549-T24 cells [180]. Autophagy-related (ATG) proteins as the main components of the autophagy process are involved in regulation of the autophagy initiation, autophagosomal maturation, lysosomal fusion, and autophagolyosomal degradation [181]. There was LINC01296 up regulation in NSCLC samples. LINC01296 promoted the NSCLC progression and paclitaxel resistance through miR-143-3p/ATG2B axis [182]. MiR-199a-5p inhibited autophagy in MDR lung tumor cells by activating the PI3K/Akt/mTOR axis, eEF2K expression, and decreasing ATG5 expression. There was miR-199a-5p up regulation in PTX resistant lung tumor cells [183]. Tumor cells can develop chemo resistance by decreasing drug absorption and facilitating drug efflux [184]. MRP1, MDR1, and BCRP belong to the ABC protein family involved in drug efflux [185]. GST-π reduces drug toxicity by binding to the hydrophobic and electrophilic compounds via glutathione reduction that result in chemo resistance [186–188]. Inhibition of miR-155 down regulated the MRP1, MDR1, GST-π, and BCRP in A549/Dox cells. MiR-155 repressing also down regulated Bcl-2 and Survivin, while up regulated CASP8 and CASP3 that enhanced apoptosis in lung tumor cells. MiR-155 inhibition also reduced AKT and ERK phosphorylation to inhibit PI3K/AKT and MAPK signaling pathways that reversed DOX resistance in lung tumor cells [189].

## Conclusions

Doxorubicin and Paclitaxel are widely used as the first line chemotherapeutic drugs in lung cancer patients. However, a significant percentage of patients show resistance to these drugs. Therefore, considering the DOX/TXL side effects in normal body tissues, it is required to introduce the novel prognostic markers to predict the Doxorubicin and Paclitaxel responses in lung cancer. The present review is an effective step towards introducing miRNAs as the non-invasive markers to predict DOX/TXL response in lung cancer which improves the therapeutic strategies to prolong the survival rates in these patients. However, the introduction of miRNAs as the non-invasive prognostic markers in lung cancer patients requires more clinical studies. In this context, it is required to assess the circulating levels of miRNAs in body fluids to clinically use them as the non-invasive markers in screening programs among lung cancer patients and healthy people with a positive familial history. Considering that the miRNAs mainly promote the sensitivity of lung tumor cells to Paclitaxel and Doxorubicin, microRNA mimics strategies can have the promising therapeutic effects in these patients. However, more animal studies and clinical trials are needed to be able to clinically use the microRNA mimics to treat the DOX/TXL-resistant lung cancer patients.

## Data Availability

The datasets used and/or analyzed during the current study are available from the corresponding author on reasonable request.

## Abbreviations

AA: Arachidonic acid
ATG: Autophagy-related
CTSL: Cathepsin L
CHK1: Checkpoint kinase 1
circRNAs: Circular RNAs
DOX: Doxorubicin
EMT: Epithelial-mesenchymal transition
ECM: Extracellular matrix
GRK5: G protein-coupled receptor kinase 5
HR: Homologous recombination
lncRNAs: Long noncoding RNAs
MMPs: Matrix metalloproteinases
miRNAs: MicroRNAs
MTAs: Microtubule targeting agents
MDM2: Mouse double minute 2 homolog
MDR: Multidrug resistance
NSCLC: Non-small cell lung cancer
PAK1: P21-activated kinase
TXL: Paclitaxel
P-gp: P-glycoprotein
ROS: Reactive oxygen species
RTKs: Receptor tyrosine kinases
